# Role and regulation of autophagy in heat stress responses of tomato plants

**DOI:** 10.3389/fpls.2014.00174

**Published:** 2014-04-30

**Authors:** Jie Zhou, Jian Wang, Jing-Quan Yu, Zhixiang Chen

**Affiliations:** ^1^Department of Horticulture, Zhejiang UniversityHangzhou, China; ^2^Department of Botany and Plant Pathology, Purdue UniversityWest Lafayette, IN, USA

**Keywords:** autophagy, heat tolerance, tomato, WRKY33, NBR1, ATG5, ATG7

## Abstract

As sessile organisms, plants are constantly exposed to a wide spectrum of stress conditions such as high temperature, which causes protein misfolding. Misfolded proteins are highly toxic and must be efficiently removed to reduce cellular proteotoxic stress if restoration of native conformations is unsuccessful. Although selective autophagy is known to function in protein quality control by targeting degradation of misfolded and potentially toxic proteins, its role and regulation in heat stress responses have not been analyzed in crop plants. In the present study, we found that heat stress induced expression of autophagy-related (ATG) genes and accumulation of autophagosomes in tomato plants. Virus-induced gene silencing (VIGS) of tomato *ATG5* and *ATG7* genes resulted in increased sensitivity of tomato plants to heat stress based on both increased development of heat stress symptoms and compromised photosynthetic parameters of heat-stressed leaf tissues. Silencing of tomato homologs for the selective autophagy receptor NBR1, which targets ubiquitinated protein aggregates, also compromised tomato heat tolerance. To better understand the regulation of heat-induced autophagy, we found that silencing of tomato *ATG5*, *ATG7*, or *NBR1* compromised heat-induced expression of not only the targeted genes but also other autophagy-related genes. Furthermore, we identified two tomato genes encoding proteins highly homologous to Arabidopsis WRKY33 transcription factor, which has been previously shown to interact physically with an autophagy protein. Silencing of tomato *WRKY33* genes compromised tomato heat tolerance and reduced heat-induced *ATG* gene expression and autophagosome accumulation. Based on these results, we propose that heat-induced autophagy in tomato is subject to cooperative regulation by both WRKY33 and ATG proteins and plays a critical role in tomato heat tolerance, mostly likely through selective removal of heat-induced protein aggregates.

## Introduction

Autophagy is a highly conserved intracellular degradation system in eukaryotes for removal and recycling of cytoplasmic components including damaged proteins and organelles (Klionsky, 2005). Central to autophagy is the formation of autophagosomes resulting from the dynamic membrane reorganization. In yeast, more than 30 autophagy-related (ATG) genes have been identified and their products often form functional groups that cooperate to perform the physiologically continuous but mechanistically distinct processes of autophagy including the induction of autophagy, autophagosome nucleation, elongation, maturation, and fusion with vacuoles (He and Klionsky, 2009). Autophagy is active at very low levels but is highly inducible in responses to stress and extracellular cues (He and Klionsky, 2009). In yeast and animal systems, the serine/threonine protein kinase TOR (target of rapamycin) functions as a central inhibitor of autophagosome formation. In yeast, inhibition of TOR leads to activation of ATG1, which can then bind ATG13 and ATG17 with increased affinities to promote assembly of the ATG1-ATG13-ATG17 scaffold and initiation of autophagosome formation through recruitment of multiple ATG proteins (He and Klionsky, 2009). The rapid increase in the autophagic flux during the early minutes or hours of exposure to stress conditions is mostly mediated by post-translational modifications of the core machinery of autophagy (He and Klionsky, 2009). A delayed and protracted autophagic response, however, also relies on activation of specific transcription programs involving stress-responsive transcription factors.

Over the past two decades or so, more than 30 ATG genes have been identified in Arabidopsis and other plants including tobacco, rice, and maize. Functional analysis of the ATG genes has shown that autophagy plays an important role in nutrient recycling and utilization in plants (Bassham et al., 2006; Liu and Bassham, 2012). Autophagy is also involved in the regulation of plant senescence, which may be considered a process of nutrient redistribution. In addition, autophagy shapes plant innate immune responses (Zhou et al., 2014b). Autophagy is also induced by a wide spectrum of abiotic stresses including oxidative, high salt, osmotic stress and heat conditions (Slavikova et al., 2008; Liu et al., 2009). Autophagy-defective mutants or transgenic plants are hypersensitive to reactive oxygen species (ROS), salt, drought, and heat conditions (Xiong et al., 2007a,b; Liu et al., 2009; Zhou et al., 2013, 2014c). As in other organisms, formation of autophagosomes and expression of *ATG* genes are induced by a variety of stresses and environmental cues in plants. TOR is also a negative regulator of autophagy in plants (Liu and Bassham, 2010). Furthermore, a NADPH oxidase inhibitor blocks autophagy induction upon nutrient starvation and salt stress, but not during osmotic stress (Liu and Bassham, 2010). Thus, ROS may mediate induction of autophagy during some, but not all stress conditions. There is, however, little information available about the transcriptional regulation of plant autophagy-associated genes under stress conditions.

In the present study, we analyze the role and regulation of autophagy in heat stress tolerance of tomato plants (*Solanum lycopersicum*). Heat is an important abiotic stress condition that can cause misfolding and denaturation of proteins. As an important protein quality control mechanism, autophagy could play a critical role in removal of those misfolded/denatured and potentially highly toxic proteins or protein aggregates that fail to be reestablished for normal protein conformations (Kraft et al., 2010; Johansen and Lamark, 2011; Shaid et al., 2013). Tomato is one of the most important vegetable plants closely related to many commercially important plants including potato, eggplant, peppers, tobacco, and petunias and an important model plant because it has a number of interesting features such as fleshy fruits not shared by other model plants (e.g., Arabidopsis and rice) and has many recognized wild species. The tomato genome has been sequenced (Consortium, 2012) and its genes can be functionally analyzed through a number of complementary approaches including virus-induced gene silencing (VIGS) (Liu et al., 2002). From the sequenced tomato genome, we have identified tomato homologs for ATG5 and ATG7, two important autophagy-related proteins that have been subjected to functional analysis in Arabidopsis (Yoshimoto, 2010; Lai et al., 2011b; Zhou et al., 2013). We have also identified tomato homologs for NBR1, a plant selective autophagy receptor (Svenning et al., 2011; Zhou et al., 2013, 2014c), and WRKY33, a transcription factor that physically interacts with ATG18a in Arabidopsis (Lai et al., 2011b). Using a variety of molecular approaches including VIGS, we analyzed the roles of these genes in heat-induced autophagy and heat stress tolerance. These experiments not only support the critical role of autophagy in plant heat tolerance but also provide new important insights into the regulation of autophagy during plant stress responses.

## Materials and methods

### Plant materials and growth conditions

Tomato (*Solanum lycopersicum* L. cv. Ailsa Craig) seeds were germinated in a growth medium filled with a mixture of peat and vermiculite (7:3, v/v) in trays in a growth chamber. When the first true leaf was fully expanded, seedlings were transplanted into plastic pots containing the same medium. The growth conditions were as follows: light/dark cycle, 22/20°C, and photosynthetic photon flux density (PPFD), 600 μmol m^−2^ s^−1^.

### Quantitative RT-PCR (qRT-PCR)

Total RNA was isolated from tomato leaves using Trizol reagent (Sangon Co., Shanghai, China), according to the manufacture's recommendations. Genomic DNA was removed with the RNeasy Mini Kit (Qiagen Co., Hilden, Germany). 1 μg RNA was reverse-transcribed using the ReverTra Ace qPCR RT Kit (Toyobo Co., Osaka, Japan), following the manufacturer's instructions. Gene-specific RT-PCR primers were designed based on their cDNA sequences (Supplemental Table S1).

The quantitative real-time PCR was performed using the iCycleri QTM real-time PCR detection system (Bio-Rad Co., Hercules, CA, USA). Each reaction (25 μL) consisted 12.5 μL of SYBR Green PCR Master Mix (Takara Co., Chiga, Japan), 1 μL of diluted cDNA and 0.1 μmol forward and reserve primers. The PCR cycling conditions and the calculation of relative gene expression were as previously described. The tomato *ACTIN* gene was used as internal control as previously described (Zhou et al., 2014a).

### Virus-induced gene silencing (VIGS)

The tobacco rattle virus (TRV) VIGS constructs for silencing of tomato *ATG5*, *ATG7*, *NBR1a*, *NBR1b*, *WRKY33a*, and *WRKY33b* genes were generated by PCR amplification using gene-specific primers (Supplemental Table S2), digested with appropriate restriction enzymes and ligated into the same sites of pTRV2. The resulting plasmids were transformed into *Agrobacterium tumefaciens* GV3101. *Agrobacterium*-mediated virus infection was performed as previously described (Ekengren et al., 2003). Plants were then kept at 22/20°C under 150 μmol m^−2^ s^−1^ PPFD for 30 days before they were used for the experiments (Kandoth et al., 2007). Leaflets in the terminal of the fifth fully expanded leaves, which showed 20–30% transcript levels of control plants, were used. Each replicate had 12 plants.

### Assessment of heat tolerance

To evaluate the role of autophagy in heat stress responses of tomato plants, tomato plants at the five-leaf stage were transferred to a growth chamber for heat stress treatment (45°C, 400 μmol m^−2^ s^−1^ PPFD, 8 h).

For determination of electrolyte leakage (EL) caused by high temperature, the leaflets in the terminal of the fifth fully expanded leaves were measured after heat stress as previous described (Huang et al., 2010). Chlorophyll fluorescence was measured using an Imaging-PAM Chlorophyll Fluorometer equipped with a computer-operated PAM-control unit (IMAG-MAXI; Heinz Walz, Effeltrich, Germany). The plants were maintained in the dark for more than 30 min before the measurements were performed. The intensities of the actinic light and saturating light were 280 and 2500 μmol mol^−2^ s^−1^ PPFD, respectively. The maximum quantum yield of PSII (Fv/Fm) was measured and calculated as previous described (Zhou et al., 2014a). Three replicates for each treatment were used with 12 plants for each replicate.

The light-saturated CO_2_ assimilation (Asat), stomatal conductance (Gs), and intracellular CO_2_ concentration (Ci) were determined in the silenced and pTRV plants with an infrared gas analyzer-based potable photosynthesis system (LI-6400; Li-COR, Lincoln, NE, USA). The air temperature, relative humidity, CO_2_ concentration, and PPFD were maintained at 22°C, 85%, 380 μmol mol^−1^, and 1000 μmol m^−2^ s^−1^, respectively.

### Separation and measurements of total and insoluble proteins

Tomato leaves were collected before and after heat treatment, ground in liquid nitrogen and homogenized in a detergent containing extraction buffer (100 mMTris/HCl, pH 8.0, 10 mM NaCl, 1 mM EDTA, 1% Triton X-100, 0.2% β-mercaptoethanol). Soluble and detergent-resistant insoluble proteins were separated through low-speed centrifugation and measured as previously described (Zhou et al., 2013).

### Visualization of induction of autophagy

For visualization of autophagosomes, tomato leaves were vacuum-infiltrated with 1 μM of the fluorescence dye LysoTracker Green DND-26 (Cell Signaling Technology, Danvers, MA, USA) or 500 μ M of mondansylcadaverine (MDC) (Sigma-Aldrich, St. Louis, MO, USA). Fluorescence was visualized using a Zeiss LSM510 UVMeta laser scanning confocal microscope (Zeiss Co., Munchen, Germany) and images were superimposed using Zeiss LSM510 software.

## Results

### Identification of tomato *ATG5*, *ATG7*, and *NBR1* genes

To analyze the role of autophagy in tomato heat tolerance, we chose first to focus on tomato *ATG5* and *ATG7* as potential targets for gene silencing as their products are required for the core process of autophagy and mutants of their Arabidopsis homologs, which are single-copy genes, have been widely used for functional analysis of autophagy (Yoshimoto, 2010; Lai et al., 2011b; Zhou et al., 2013). From the sequenced tomato genome, we identified two tomato ATG5 genes, *ATG5a* (Sl02g038380) and *ATG5b* (Sl06g043140). Based on its genomic and full-length cDNA sequences, *ATG5a* has an intron-exon structure similar to that of Arabidopsis *ATG5* with eight exons and seven introns (Supplemental Figure 1A). *ATG5a* encodes a protein containing 369 amino acid residues and sharing approximately 60% sequence identity with Arabidopsis ATG5. The genomic sequence of tomato *ATG5b* is 97% identical to the coding sequence of tomato ATG5a (Supplemental Figure 1B). The lack of introns in tomato *ATG5b* indicates that the gene was copied from mRNA of tomato *ATG5a* and incorporated into the tomato genome. Further sequence analysis revealed multiple mutations in tomato *ATG5b*, including a G to A transition at nucleotide position 246 that changes a tryptophan to a stop codon, resulting in a predicted loss of the last 277 amino acids (77%) of the polypeptide if the mutation is not removed by alternative splicing (Supplemental Figure 2). Thus, tomato *ATG5b* likely encodes a nonfunctional protein even if it is expressed in tomato tissues. Like Arabidopsis, tomato contains a single *ATG7* gene (Sl11g068930) that encodes a protein of 715 amino acid residues. Tomato ATG7 shares approximately 70% sequence identity with Arabidopsis ATG7.

**Figure 1:**
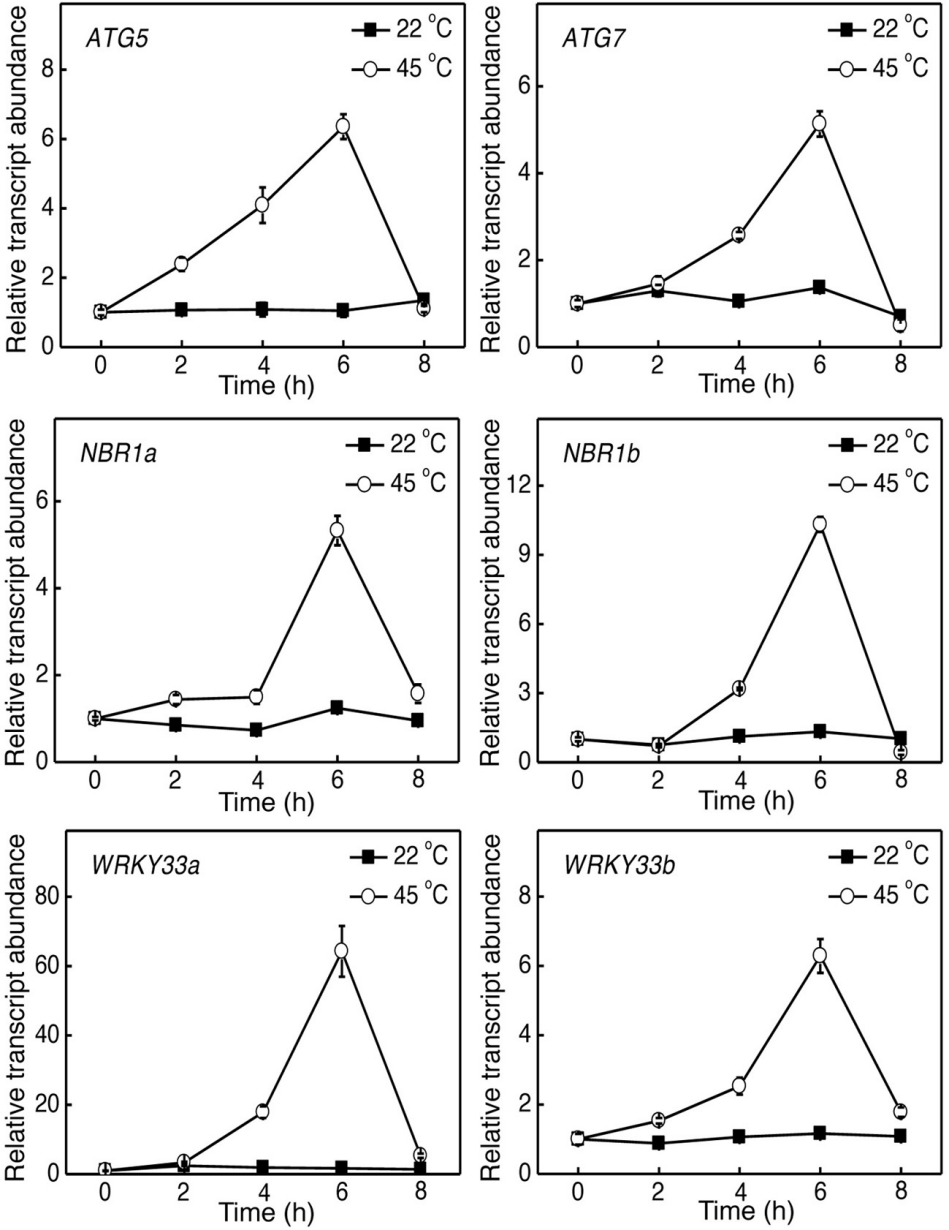
**Induction of tomato *ATG5*, *ATG7*, *NBR1*, and *WRKY33* genes by heat stress**. Six weeks-old tomato plants were placed in the 22 and 45°C growth chambers and total RNA was isolated from leaf samples collected at indicated times. Transcript levels were determined using real-time qRT-PCR. Error bars indicate SE (*n* = 3).

There is a mounting body of evidence that selective autophagy of dysfunctional organelles and toxic macromolecules mediated by selective autophagy receptors play a critical role in protein/organelle quality control and in responses to adverse environmental and physiological conditions (Kraft et al., 2010; Johansen and Lamark, 2011; Floyd et al., 2012; Shaid et al., 2013). Previously Arabidopsis NBR1, homolog of the mammalian autophagy receptors P62 and NBR1, has been analyzed and found to play a critical role in plant responses to a spectrum of abiotic stresses by targeting stress-induced cytosolic protein aggregates (Zhou et al., 2013). Unlike in Arabidopsis, which contains a single *NBR1* gene, tomato contains two *NBR1* genes, *NBR1a* (Sl03g112230), and *NBR1b* (Sl06g071770), with intron-exon structures similar to each other and to that of Arabidopsis *NBR1*. Tomato *NBR1a* and *NBR1b* encodes proteins of 864 and 737 amino acids, respectively (Supplemental Figure 3), which share approximately 50% sequence identify with each other and with that of Arabidopsis NBR1. Like Arabidopsis NBR1, both tomato NBR1a and NBR1b contain two highly conserved ubiquitin-associated (UBA) domains and a WxxI ATG8-interacting motif at their respective C-terminus (Supplemental Figure 3).

### Heat induction of autophagy-related genes in tomato

To determine the role of autophagy in tomato heat tolerance, we firstly analyzed the effect of heat stress on expression of tomato *ATG5*, *ATG7*, and *NBR1* genes. Tomato seedlings were placed in the 22 and 45°C chambers, and the *ATG* and *NBR1* gene transcripts were analyzed by qRT-PCR using total RNA isolated from the leaflets of the fifth fully expanded leaves. As shown in Figure 1, the levels of tomato *ATG5, ATG7* and *NBR1* transcripts remained unchanged at 22°C throughout the 8-h period of the experiments. In contrast, transcript levels of the autophagy genes were elevated after 2–4 h of heat stress (45°C) and displayed large increases after 6 h of heat stress (Figure 1). After 6-h heat stress, the transcript levels of the *ATG* and *NBR1* genes started to decline and reduced to basal levels by 8 h at 45°C, when the leaves of seedlings started to show symptoms of dehydration (Figure 1). Thus, heat stress induced the expression of autophagy-related genes.

### Silencing of tomato *ATG5*, *ATG7*, and *NBR1* genes

**Figure 2:**
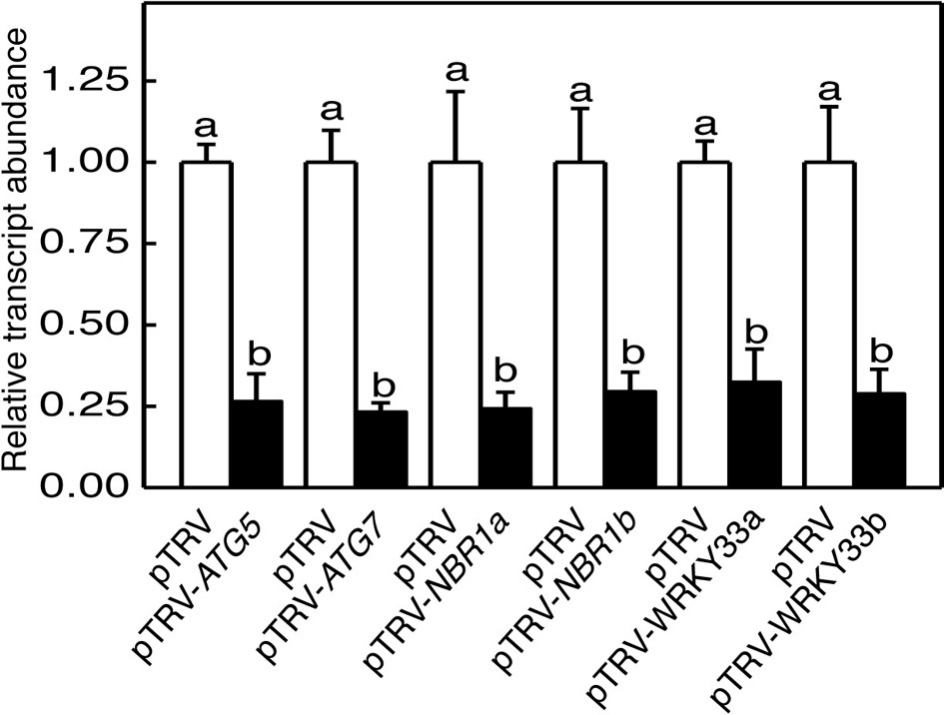
**TRV-mediated silencing of tomato *ATG5*, *ATG7*, *NBR1*, and *WRKY33* genes**. Transcript levels for each silenced gene in tomato plants infiltrated with *Agrobacterium* cells harboring the empty pTVR vector or the corresponding silencing vector were determined by qRT-PCR analysis using total RNA isolated from the terminal leaflets of the fifth leaves of *Agrobacterium*-infiltrated tomato plants. Error bars indicate SE (*n* = 3). According to Duncan's multiple range test (*P* = 0.05), means of lesion areas do not differ significantly if they are indicated with the same letter.

To determine directly the role of autophagy in tomato responses to heat stress, we used VIGS to assess the impact of down-regulated expression of tomato *ATG5*, *ATG7*, *NBR1a*, and *NBR1b* on tomato heat tolerance. Gene-specific DNA fragments were cloned into the pTRV vector and *Agrobacterium* cells harboring the VIGS vectors were infiltrated into tomato cotyledons. We used qRT-PCR to compare the transcript levels for tomato *ATG5*, *ATG7*, *NBR1a*, and *NBR1b* in tomato plants infiltrated with the pTRV empty vector or infiltrated with the pTRV-*ATG5*, pTRV-*ATG7*, pTRV-*NBR1a*, or pTRV-*NBR1b* silencing vector. As shown in Figure 2, the basal transcript levels of *ATG5*, *ATG7*, *NBR1a*, and *NBR1b* were unchanged in tomato plants after infiltration with the pTRV empty vector but decreased 70–80% in the leaves of plants after infiltration with their respective silencing vectors (Figure 2). No significant alteration in growth or development was observed upon silencing of the *ATG5*, *ATG7*, *NBR1a*, or *NBR1b* gene.

**Figure 3:**
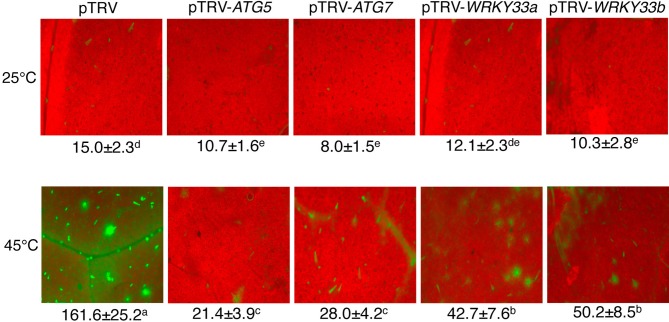
**Detection of autophagic activity using LysoTracker Green**. Comparison of tomato plants infiltrated with *Agrobacterium* cells harboring the empty pTVR vector or a silencing vector for an indicated gene in terms of LysoTracker Green fluorescence signals. The plants were placed into the 22 and 45°C growth chambers and analyzed after 6-h treatment. Numbers of punctate LysoTracker Green fluorescence spots per 10,000 μm^2^ section from the cells in the central areas of the terminal leaflets of the fifth leaves were indicated below the images. Means and SE were calculated from three experiments. According to Duncan's multiple range test (*P* = 0.05), means of lesion areas do not differ significantly if they are indicated with the same letter.

Abiotic stress including high temperature induces both ATG gene expression and formation of autophagosomes. To further assess the effect of silencing of tomato *ATG5* and *ATG7* on heat-induced autophagy, we used LysoTracker Green dye as a probe to detect autolysosome-like structures. The LysoTracker dye has been widely used as a probe for detecting autophagic activity in a variety of organisms including plants (Otegui et al., 2005). Comparative studies with other autophagosome makers such as ATG8 have shown that although LysoTracker dyes stain acidic organelles, including autophagosomes, up-regulated LysoTracker-stained structures are biologically characteristic of induced autophagic activity (Phadwal et al., 2012; Chikte et al., 2014). Under the normal temperature (22°C), we observed low numbers of punctate green fluorescent signals in both pTRV and gene silenced plants (Figure 3). After 6-h heat stress, however, the numbers of punctate green fluorescent signals increased by more than 10 fold in the control plants infiltrated with the pTRV empty vector (Figure 3). Importantly, in the plants infiltrated with the pTRV-*ATG5* or pTRV-*ATG7* silencing vector, there was only a 2–3-fold increase in the numbers of the punctate fluorescence signals after 6-h heat stress (Figure 3).

**Figure 4:**
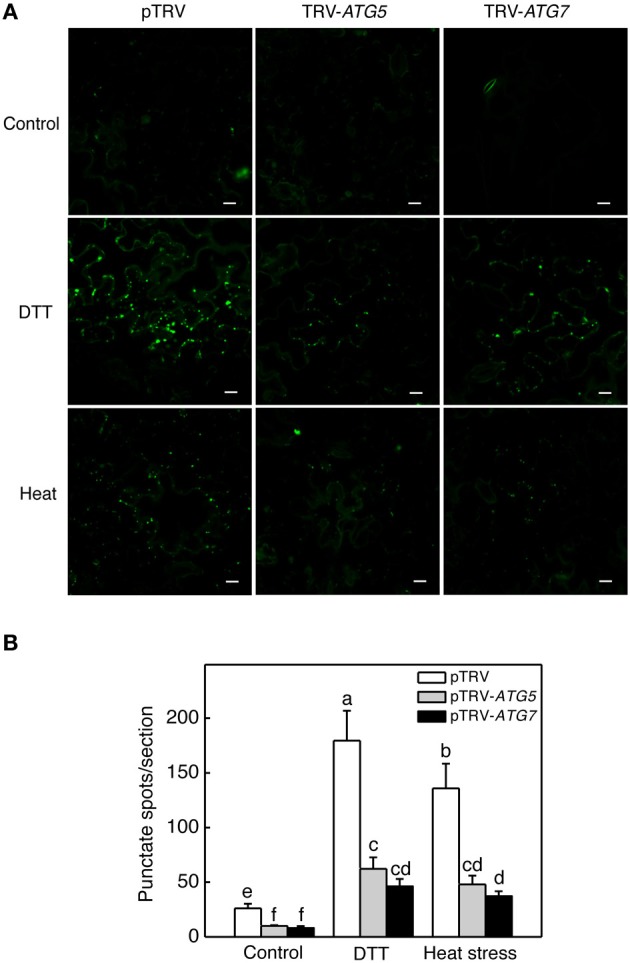
**MDC-stained autophagosomes in tomato leaves. (A)** Comparison of tomato plants infiltrated with *Agrobacterium* cells harboring the empty pTVR vector or a silencing vector for an indicated gene in terms of MDC fluorescence signals. The plants were placed into the 22 and 45°C growth chambers and analyzed after 6-h treatment. DTT (2 mM, 6 h) treatment was included as positive control. **(B)** Numbers of punctate MDC fluorescence spots per 10,000 μm^2^ section were indicated. Means and SE were calculated from three experiments. According to Duncan's multiple range test (*P* = 0.05), means of lesion areas do not differ significantly if they are indicated with the same letter. Bars = 10 μm.

We also used MDC as a probe for detection of autophagic activity in tomato leaves. MDC is an autofluorescent dye that stains autophagosomes in mammals and plants (Biederbick et al., 1995; Munafo and Colombo, 2001; Contento et al., 2005; Liu and Bassham, 2010). Under the normal temperature (22°C), again, we observed low numbers of punctate fluorescent signals in both control (pTRV) and *ATG5*- or *ATG7*-silenced plants (Figure 4). After treatment with dithiothreitol (DTT), a known inducer of autophagy (Liu et al., 2012), the numbers of punctate fluorescent signals increased by more than 8 fold in control plants infiltrated with the pTRV empty vector (Figure 4). After 6-h heat treatment, the numbers of punctate fluorescent signals also increased by about 6 fold in control plants infiltrated with the pTRV empty vector (Figure 4). In the plants infiltrated with the pTRV-*ATG5* or pTRV-*ATG7* silencing vector, the numbers of the punctate fluorescence signals after DTT or heat stress were substantially reduced when compared to those in DTT- or heat-treated control plants (Figure 4). These observations confirmed that heat-induced autophagy was partially blocked by silencing of the tomato *ATG5* and *ATG7* genes.

### Compromised heat tolerance of autophagy-silenced tomato plants

**Figure 5:**
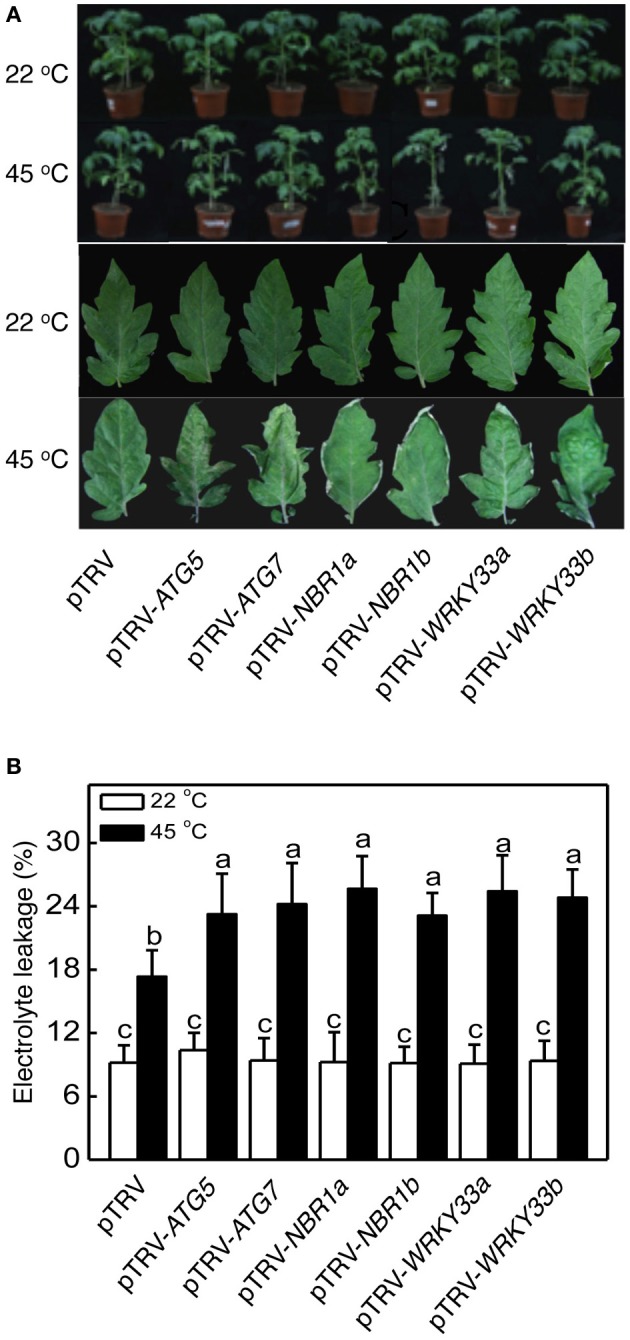
**Functional analysis of tomato *ATG5, ATG7, NBR1*, and *WRKY33* in tomato heat tolerance using TRV-mediated gene silencing. (A)** Tomato plants infiltrated with *Agrobacterium* cells harboring the empty pTVR vector or the silencing pTRV-*ATG5*, pTRV-*ATG7*, pTRV-*NBR1*, or pTRV-*WRKY33* vector were placed in a 22 or 45°C growth chamber for 8 h. The pictures of the whole plants (upper panel) or the terminal leaflets of the fifth leaves (lower panel) were taken after 3-day recovery. **(B)** Electrolyte leakage (EL) of the terminal leaflets of the fifth leaves were determined immediately after 8 h at 22 or 45°C heat treatment. Means and SE were calculated from average EL values determined from three experiments with 10 leaves per experiment for each genotype. According to Duncan's multiple range test (*P* = 0.05), means of lesion areas do not differ significantly if they are indicated with the same letter.

For comparison of heat tolerance of pTRV, pTRV-*ATG5*, pTRV-*ATG7*, pTRV-*NBR1a*, and pTRV-*NBR1b* plants, they were placed in a 45°C growth chamber for 8 h and then moved to room temperature for 3-day recovery. For heat–treated pTRV control plants, only patches of old leaves displayed symptoms of dehydration while a majority of the leaves remained green and viable after recovery (Figure 5A). On the other hand, a majority of fully expanded leaves from the pTRV-*ATG5*, pTRV-*ATG7*, pTRV-*NBR1a*, and pTRV-*NBR1b* plants exhibited wilting after the recovery (Figure 5A). The more severe symptoms in autophagy-suppressed tomato plants after heat stress were confirmed by increased EL in the silenced plants relative to that in the pTRV control plants (Figure 5B).

**Figure 6:**
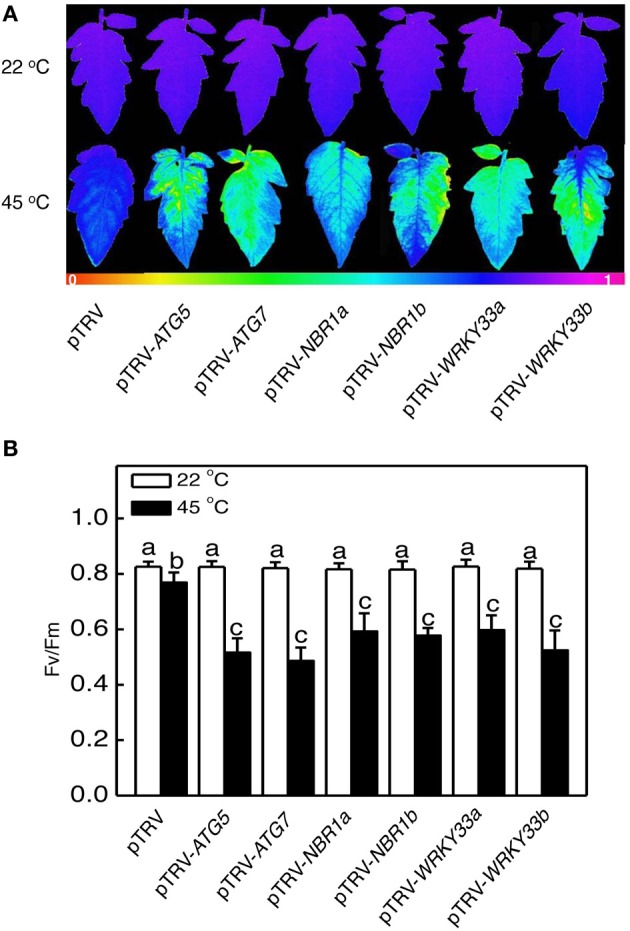
**The effect of heat stress on the efficiency of PSII photochemistry**. Fv/Fm images **(A)** and values **(B)** of the terminal leaflets of the fifth leaves were determined immediately after 8 h at 22 or 45°C heat treatment. The color code in the images ranged from 0 (black) to 1.0 (purple). Means and SE were calculated from average Fv/Fm values determined from three experiments with 10 leaves per experiment for each type of plants. According to Duncan's multiple range test (*P* = 0.05), means of lesion areas do not differ significantly if they are indicated with the same letter.

**Figure 7:**
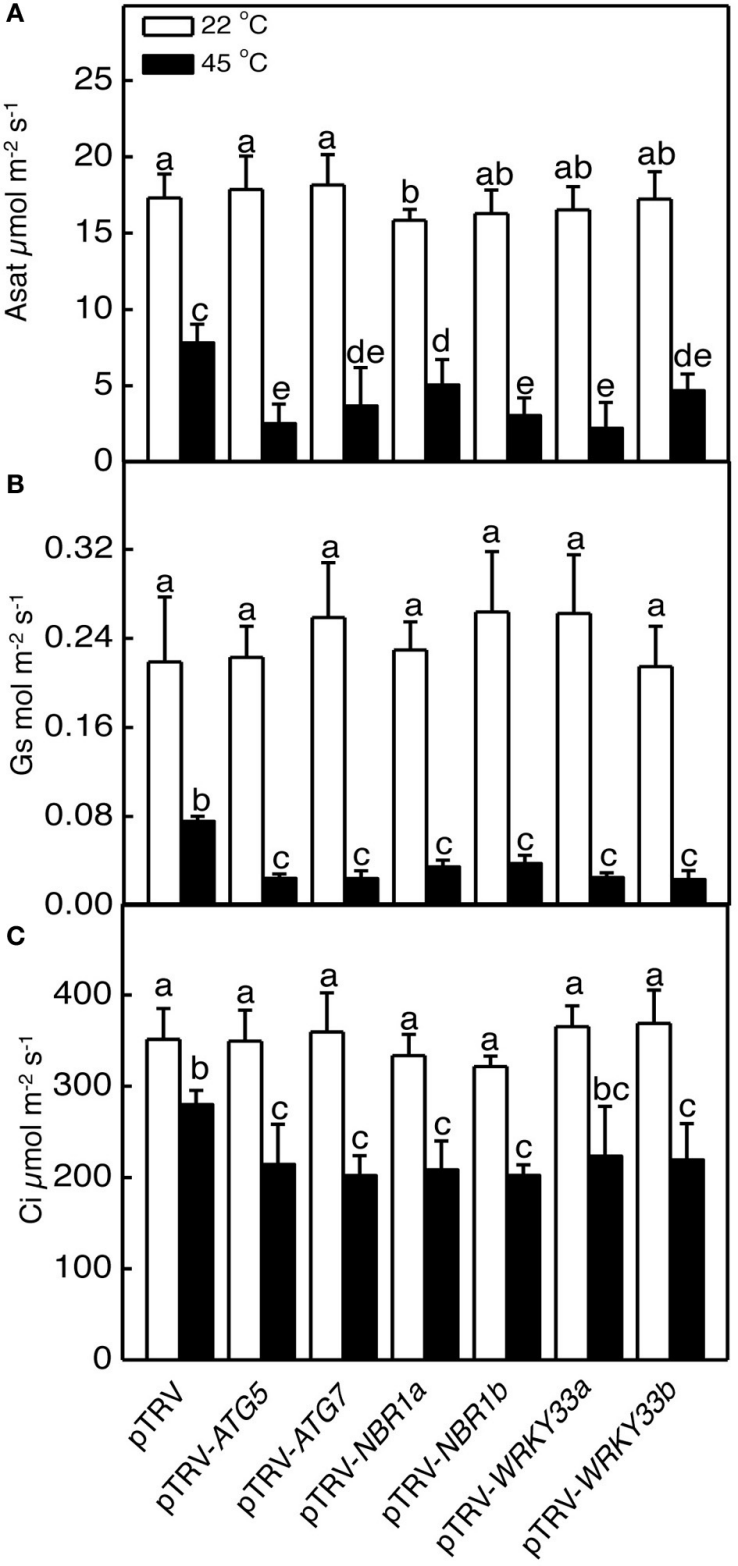
**The effect of heat stress on the capacity of photosynthesis**. Light-saturated CO_2_ assimilation rate (Asat) **(A)**, stomatal conductance (Gs) **(B)** and intracellular CO_2_ concentration (Ci) **(C)** were determined following 1-day recovery after heat stress. Means and SE were calculated from average values determined from three experiments with 10 leaves per experiment for each type of plants. According to Duncan's multiple range test (*P* = 0.05), means of lesion areas do not differ significantly if they are indicated with the same letter.

Heat has a harmful effect on various biology processes including photosynthesis. To further investigate responses of *ATG5*-, *ATG7*-, and *NBR1*-silenced tomato plants to heat stress, we compared these tomato plants with the pTRV control tomato plants for the effects of heat stress on the maximum quantum yield of photosystem II (PSII) (Fv/Fm) of leaves immediately after heat treatment and light-saturated CO_2_ assimilation rate (Asat) following 1-day recovery after heat stress. As shown in Figure 6, after 8-h at 45°C, Fv/Fm values for silencing plants were 22–37% lower than those of control pTRV plants. Likewise, the Asat values for the silenced plants were 35–68% lower than those of pTRV control plants when assayed after 1-day recovery following heat stress (Figure 7). In addition, *ATG5*-, *ATG7*-, and *NBR1*-silenced tomato plants had reduced stomatal conductance (Gs) and intracellular CO_2_ concentration (Ci) compared to the unsilenced pTRV control plants after heat stress (Figure 7). Thus, photosynthetic efficiency and capacity were more compromised by heat stress in the autophagy-suppressed tomato plants than in the control plants.

### Increased accumulation of insoluble proteins in autophagy-silenced plants

**Figure 8:**
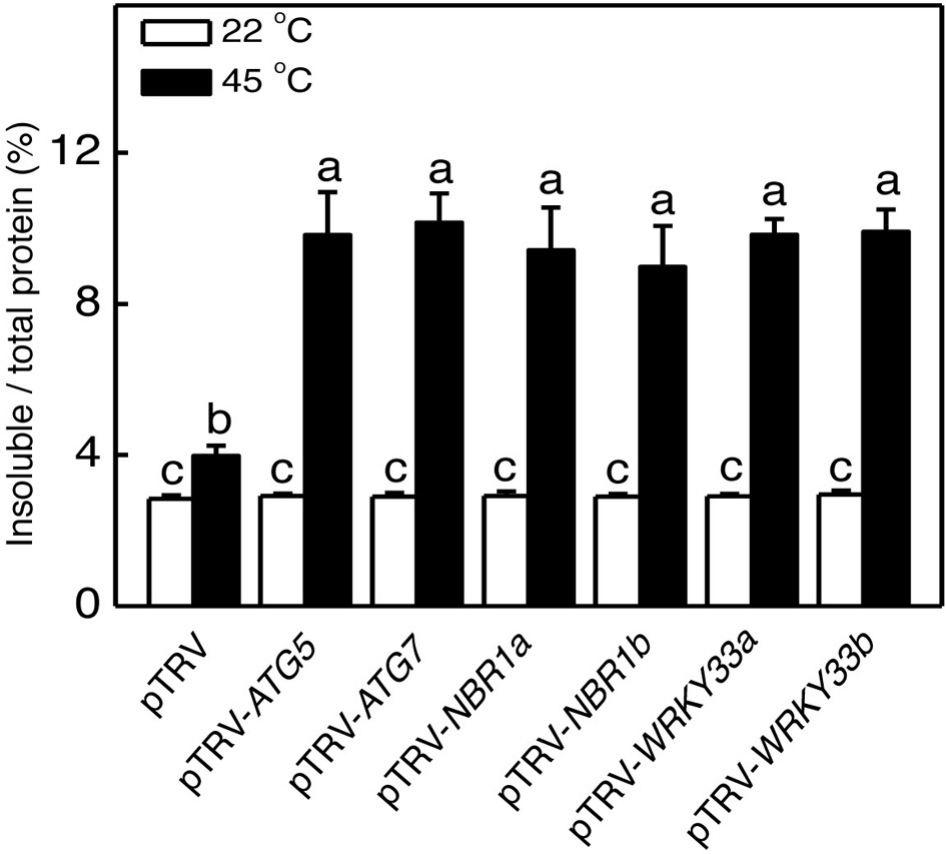
**Accumulation of insoluble protein aggregates under heat stress**. Leaf tissues from tomato plants infiltrated with *Agrobacterium* cells harboring the empty pTVR vector or the silencing pTRV-*ATG5*, pTRV-*ATG7*, pTRV-*NBR1*, or pTRV-*WRKY33* vector collected at indicated hours (h) under 45°C for preparation of total, soluble and insoluble proteins as described in Materials and Methods. Total proteins in the starting homogenates and insoluble proteins in the last pellets were determined the percentages of insoluble proteins to total proteins were calculated.

Heat stress causes protein misfolding and denaturation, which can result in formation of protein aggregates and proteotoxic stress. To analyze the role of autophagy in protection against heat-induced proteotoxic stress, we investigated the accumulation of insoluble, detergent-resistant proteins in the pTRV, pTRV-*ATG5*, pTRV-*ATG7*, pTRV-*NBR1a*, and pTRV-*NBR1b* plants after 8 h heat treatment. Total proteins were first isolated and insoluble proteins were separated by low speed centrifugation. As shown in Figure 8, the percentages of insoluble to total proteins were similar in all plants when they were grown at 22°C. After 8-h heat stress, insoluble proteins as percentages to total proteins increased only by 40% in unsilenced pTRV control plants but increased by 138–154% in the pTRV-*ATG5*, pTRV-*ATG7*, pTRV-*NBR1a*, and pTRV-*NBR1b* plants (Figure 8). By the end the heat stress, the levels of insoluble proteins in the *ATG5*-, *ATG7*-, and *NBR1*-silenced tomato plants were more than two times higher than those in the unsilenced control plants (Figure 8).

### Identification and functional analysis of tomato WRKY33 in heat tolerance

*Arabidopsis* WRKY33 is a transcription factor important for plant resistance to necrotrophic fungal pathogens and for plant heat tolerance (Zheng et al., 2006; Li et al., 2011). Arabidopsis WRKY33 interacts with Arabidopsis ATG18a, a critical component of autophagy, and plays a positive role in pathogen-induced *ATG18a* expression and autophagosome formation (Lai et al., 2011a). These results suggest that the critical role of WRKY33 in plant responses to biotic and abiotic stresses may be at least in part mediated through its positive regulation of pathogen/stress-induced autophagy. To investigate whether tomato contains similar WRKY transcription factor(s) with a critical role in heat tolerance and regulation of stress-induced autophagy, we searched the sequenced tomato genomes and identified two close WRKY33 homologs, WRKY33a (Sl09g014990) and WRKY33b (Sl06g066370). As shown in Supplemental Figure 4, Arabidopsis WRKY33, tomato WRKY33a and WRKY33b all belong to Group I WRKY transcription factors containing two WRKY domains with highly conserved amino acid sequences. High sequence similarities are also found in the N-terminal domains including the highly conserved SP clusters as putative MAPK phosphorylation sites and the intervening sequences between the two WRKY domains (Supplemental Figure 4). Furthermore, both tomato WRKY33a and WRKY33b contain a segment of about 100 amino acid residues on the C-terminal side of the second WRKY domain with substantial sequence homology with Arabid0psis WRKY33, which are absent in other related Group I WRKY transcription factors such as Arabidopsis WRKY25 and WRKY26 (Supplemental Figure 4) (Lai et al., 2011a).

To analyze heat-induced expression of tomato *WRKY33* genes, we analyzed their transcripts in the tomato seedlings grown at 22 or 45°C. As shown in Figure 1, the transcript levels of both tomato *WRKY33a* and *WRKY33b* remained low throughout the 8-h period of the experiments at 22°C. At 45°C, however, the transcript levels of tomato *WRKY33a* and *WRKY33b* were elevated with similar kinetics (Figure 1). Transcripts levels for both genes displayed substantial increases after 4-h exposure to 45°C and peaked after 6-h heat stress (Figure 1). Like those of other analyzed autophagy-related genes, the transcript levels for both *WRKY33a* and *WRKY33b* declined after 6-h heat exposure and approached those of control plants by 8-h heat exposure (Figure 1).

To determine directly the roles of tomato *WRKY33* genes, we used VIGS technology to assess the impact of their down-regulated expression on tomato heat tolerance. Tomato *WRKY33*-specific DNA fragments were cloned into the pTRV vector and *Agrobacterium* cells harboring the VIGS vectors were infiltrated into tomato leaves. As shown in Figure 2, basal expression of *WRKY33a* or *WRKY33b* was observed in the tomato plants infiltrated with the pTRV empty vector. By contrast, infiltration with either pTRV-*SlWRKY33a* or pTRV-*SlWRKY33b* silencing vector resulted in approximately 5–7-fold reduction in the transcript levels for both tomato *WRKY33a* and *WRKY33b* (Figure 2). The cross silencing likely resulted from the high sequence homology between the two genes, which share more than 75% nucleotide sequence identify. The tomato plants silenced for *WRKY33a* and *WRKY33b* were normal in growth and development and displayed no detectable morphological phenotype.

We analyzed the impact of silencing of *WRKY33* genes on tomato heat tolerance. Both control and silenced plants were placed in a 45°C growth chamber for 8 h and then moved to room temperature for 3-day recovery. Unlike heat–treated pTRV control plants, which had only some patches of old leaves that displayed symptoms of dehydration, a majority of leaves from the pTRV-*WRKY33a* and pTRV-*WRKY33b* plants exhibited extensive wilting or even bleaching after the recovery (Figure 5). Thus, silencing tomato *WRKY33* genes caused increased sensitivity to heat stress. Assays of EL, the maximum quantum yield of PSII (Fv/Fm), light-saturated CO_2_ assimilation rate (Asat), stomatal conductance (Gs), and intracellular CO_2_ concentration (Ci) confirmed that silencing of tomato *WRKY33a* and *WRKY33b* compromised tomato heat tolerance (Figures 5–7). Furthermore, silencing of tomato *WRKY33a* and *WRKY33b* led to increased accumulation of insoluble proteins under heat stress (Figure 8).

### Regulation of heat-induced autophagy by *WRKY33* and autophagy proteins

To investigate whether the critical role of tomato *WRKY33* genes in heat tolerance is associated with their positive roles in regulation of heat-induced autophagy, we analyzed whether silencing the *WRKY33* genes in tomato compromised heat-induced autophagosome formation. As shown in Figure 3, while there was a more than 10 fold increase in the numbers of punctate green fluorescent signals after 6-h heat stress in the pTRV control plants, there was only 3–5-fold increase in the pTRV-*WRKY33a* and pTRV-*WRKY33b* plants. As a result of combined reduction of both basal and induced autophagosome formation, the levels of autophagosomes in the WRKY33-silenced plants were only about 25–30% of those in the control plants after 6-h heat stress. Thus, tomato WRKY33 proteins play a positive role in heat-induced autophagosome formation.

**Figure 9:**
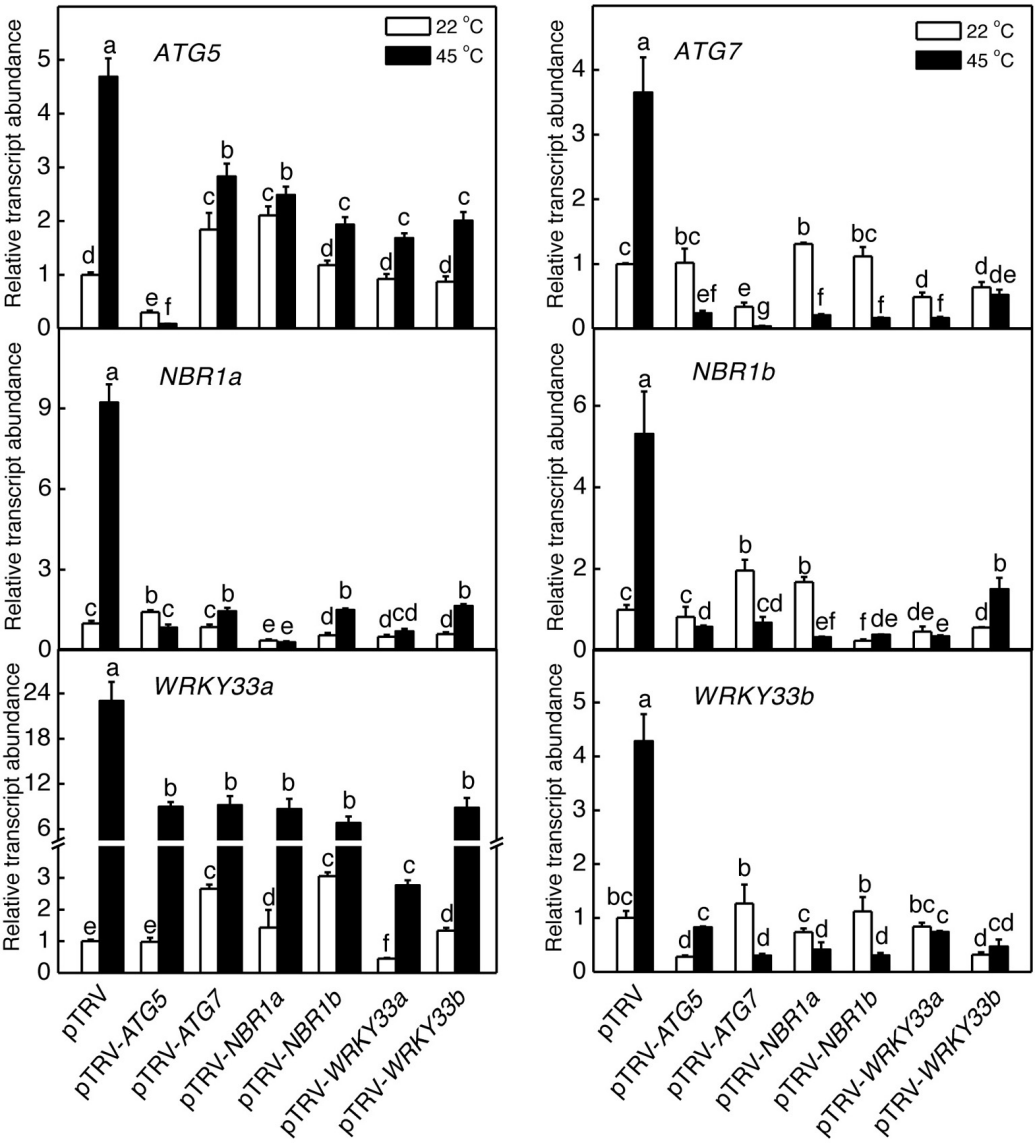
**Regulation of heat-induced expression of tomato *ATG5*, *ATG7*, *NBR1*, and *WRKY3*3 genes**. Tomato plants infiltrated with *Agrobacterium* cells harboring the empty pTVR vector or the silencing pTRV-*ATG5*, pTRV-*ATG7*, pTRV-*NBR1*, or pTRV-*WRKY33* vector were placed in a 45°C growth chamber and total RNA was isolated from leaf samples collected after 6-h heat stress for determination of transcript levels of indicated genes by qRT-PCR. Error bars indicate SE (*n* = 3). According to Duncan's multiple range test (*P* = 0.05), means of lesion areas do not differ significantly if they are indicated with the same letter.

**Figure 10:**
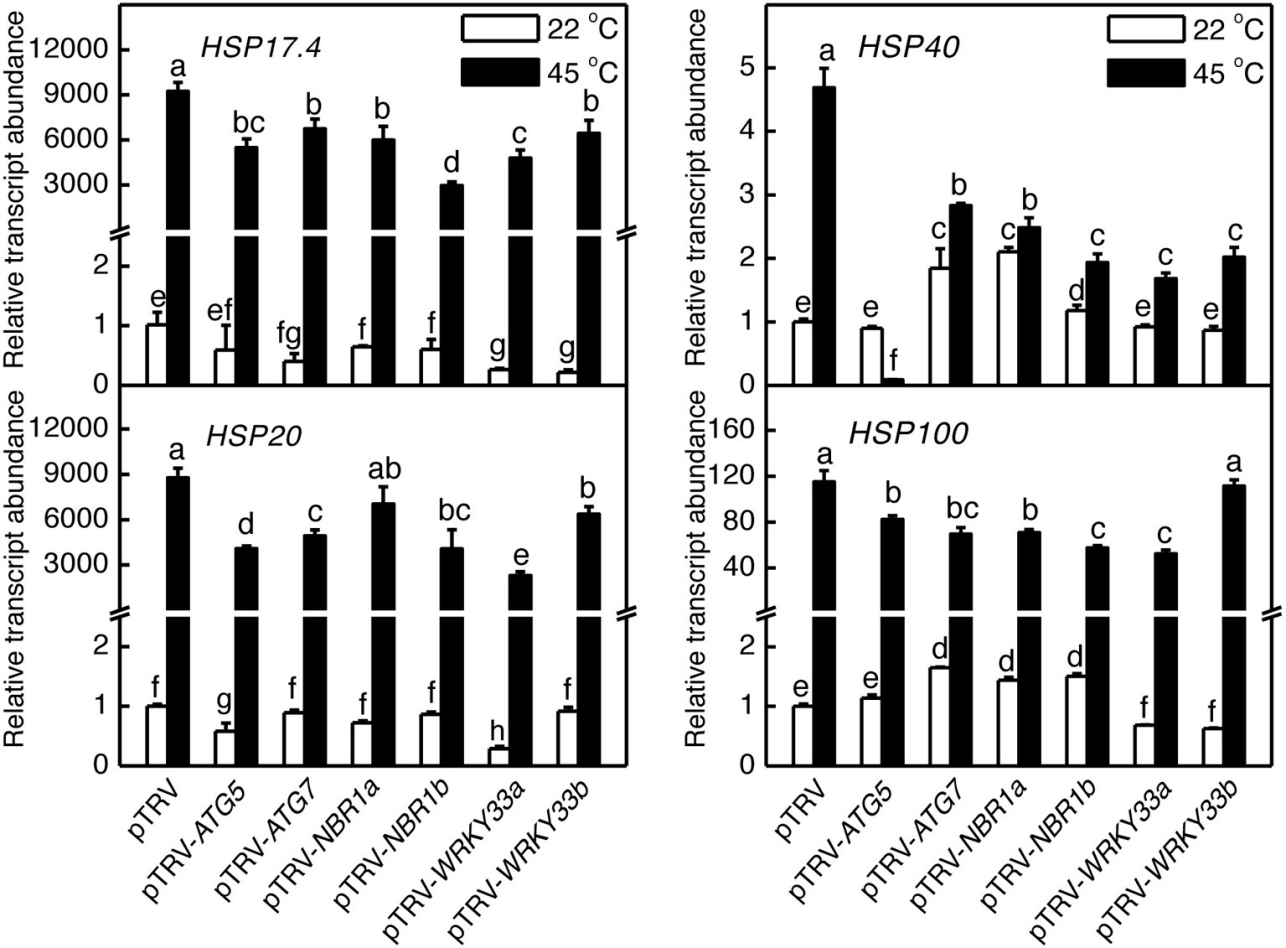
**Regulation of heat-induced expression of tomato *HSP* genes**. Tomato plants infiltrated with *Agrobacterium* cells harboring the empty pTVR vector or the silencing pTRV-*ATG5*, pTRV-*ATG7*, pTRV-*NBR1*, or pTRV-*WRKY33* vector were placed in a 45°C growth chamber and total RNA was isolated from leaf samples collected after 6-h heat stress for determination of transcript levels of indicated genes by qRT-PCR. Error bars indicate SE (*n* = 3). According to Duncan's multiple range test (*P* = 0.05), means of lesion areas do not differ significantly if they are indicated with the same letter.

We also analyzed the mutual regulation among the silencing tomato *ATG*5, *ATG7*, *NBR1*, and WRKY33 genes during plant responses to heat stress. For this purpose, we analyzed the transcript levels of *ATG5*, *ATG7*, *NBR1a*, *NBR1b*, *WRKY33a*, and *WRKY33b* in the silencing plants after 6-h heat stress. In pTRV control plants, as expected, the transcript levels for *ATG5*, *ATG7*, *NBR1a*, *NBR1b*, *WRKY33a*, and *WRKY33b* were elevated under heat stress. However, induction of these genes was all reduced not only in the plants harboring their respective silencing vectors but also in the plants harboring silencing vectors for the other genes (Figure 9). Induction of three heat shock proteins (*HSP17.6*, *HSP20*, and *HSP100*) in *ATG5-, ATG7*-, *NBR1*-, or *WRKY33*-silencing plants or pTRV plants was almost as strong as that in the pTRV control plants after 6-h heat stress (Figure 10). However, induction of a tomato *HSP40* gene was significantly compromised by silencing of the autophagy-related or WRKY33 genes (Figure 10). These results indicated that ATG and WRKY33 proteins have a positive role in heat-induced expression of autophagy-related genes.

## Discussion

In the present study, we analyzed the role of autophagy in responses to heat stress in tomato, an important horticultural crop. Both the expression of *ATG5* and *ATG7* genes and formation of autophagosomes were induced in heat-stressed tomato plants (Figures 1, 3, 4). The heat tolerance of autophagy-suppressed tomato plants due to silencing of *ATG5* and *ATG7* genes was compromised based on their increased morphological symptoms associated with enhanced defects in the efficiency and capacity of photosynthesis after heat stress (Figures 5–7). These results indicate that autophagy plays an important role in tomato heat tolerance.

Heat stress causes protein misfolding and denaturation. Misfolded/denatured proteins are highly toxic due to nonspecific binding to a variety of cellular constituent and, therefore, must be efficiently removed to prevent proteotoxic stresses (Hightower, 1991). Extensive studies in yeast and animal organisms have revealed that misfolded proteins are recognized by the protein quality control system, ubiquitinated by chaperone-dependent E3 ubiquitin ligases such as the C-terminus of Hsc70-interacting protein (CHIP) and subjected to degradation by the ubiquitin proteasome system (UPS) (Kraft et al., 2010; Shaid et al., 2013). Very recently we have conducted comprehensive genetic analysis of Arabidopsis CHIP E3 ubiquitin ligase and discovered its critical role in plant responses to a spectrum of abiotic stresses including heat stress (Zhou et al., 2014c). Previously, it has also been reported that Arabidopsis CHIP E3 ubiquitin ligase and Hsc70-4 mediate plastid-destination precursor degradation through UPS when the import of the precursors are blocked in a plastid-import mutant (Lee et al., 2009). In the present study, we demonstrated that silencing of tomato genes encoding *NBR1a* and *NBR1b*, two close homologs of mammalian ubiquitin-binding autophagy receptors P62 and NBR1, also compromised tomato heat tolerance (Figures 5–7). Thus, NBR1-mediated selective autophagy is critical in tomato heat tolerance most likely through its activity in removing heat-induced misfolded proteins. For degradation by UPS, proteins must be unfolded to enter the narrow central cavity of its barrel-shaped 20S proteolytic core since the steric conditions of a folded protein would not be able to pass through the entrance channel. Under heat stress, misfolded, or denatured proteins may form protein aggregates that are difficult to dissociate or unfold. These protein aggregates are likely to be targeted by NBR1-mediated selective autophagy if they fail to be processed by UPS. Consistent with this interpretation, compromised heat tolerance of NBR1-silenced tomato plants was associated with increased accumulation of protein aggregates under heat stress (Figure 8). These results provide further support that UPS and NBR1-mediated selective autophagy function in cooperation in the removal of misfolded proteins for protection against proteotoxic stress under adverse environmental and physiological conditions (Zhou et al., 2014c).

Heat stress induced both LysoTracker or MDC-stained autolysosome-like structures (Figures 3, 4) and expression of *ATG* and *NBR1* genes (Figure 1). Interestingly, silencing of an *ATG* or *NBR1* gene in tomato plants led to down regulation of not only the silenced gene but also other sequence-unrelated autophagy genes (Figure 9). Since ATG5, ATG7, and NBR1 proteins are not known to be regulators of gene transcription, their effect on the expression of other genes is probably indirect and most likely related to induced autophagy under heat stress. It is possible that induced autophagy under heat and perhaps other stress conditions as well has a positive role in the upregulation of autophagy genes. Consistent with the potential signaling role of autophagy, silencing of Arabidopsis *TOR* gene leads to not only constitutive formation of autophagosomes but also induced expression of some *ATG* genes (Liu and Bassham, 2010). We have also previously observed that in autophagy-deficient mutants, induction of jasmonate-regulated *PDF1.2* gene by Botrytis infection was compromised (Lai et al., 2011b). Likewise, in the *ATG5*- or *ATG7*-silenced tomato mutants, induction of a gene encoding a *HSP40* was significantly reduced (Figure 10), indicating that induced autophagy, perhaps through formation and turnover of autophagosomes, has a positive role in up-regulation of not only autophagy genes but also other genes associated with defense and stress responses. We have previously shown that in Arabidopsis *atg5* and *atg7* mutants, the protein levels of NBR1 increased greatly under heat stress (Zhou et al., 2013). Other studies have also showed that some of the autophagy-related proteins and NBR1 are themselves autophagy substrates (Svenning et al., 2011) that undergo rapid turnover and must be replenished through increased synthesis for sustained autophagy. On the other hand, when autophagy is suppressed or blocked as in the *ATG5*- and *ATG7*-silenced plants, there would be no need for increased transcription of the *ATG* or *NBR1* genes since their degradation by autophagy is inhibited.

Arabidopsis WRKY33 transcription factor plays a critical role in plant resistance to necrotrophic fungal pathogens and in plant tolerance to heat stress (Zheng et al., 2006; Li et al., 2011). Autophagy is induced by necrotrophic pathogens, heat, and salt stresses and plays an important role in plant responses to these biotic and abiotic stresses as well. We have previously shown that in the Arabidopsis *wrky33* mutants, increased formation of autophagosomes was observed in Botrytis-infected lesion areas but not in the areas surrounding the lesions found in wild-type plants (Lai et al., 2011b). In addition, induction of *ATG18a* was normal at 1 day post Botrytis infection (dpi) but was severely compromised at 2, 3, and 4 dpi in *wrky33* (Lai et al., 2011b). Thus, WRKY33 is dispensable for early induction of authophagy but necessary for sustained induction of autophagy in Botrytis-infected plants. Likewise, silencing of tomato *WRKY33* compromised heat-induced autophagy gene expression and reduced autophagosome formation (Figures 3, 8). Thus, it is likely that the critical role of WRKY33 in plant disease resistance and stress tolerance is, at least in part, mediated by its critical role in induction of autophagy.

In Arabidopsis, WRKY33 is subjected to dual-level regulation by the mitogen protein kinase 3 and 6 (MPK3/6) cascade (Mao et al., 2011). Upon pathogen infection, WRKY33 is phosphorylated by the pathogen/stress-induced MPK3/MPK6 and phosphorylation of WRKY33 is likely to promote the transcription activity of WRKY33, which can bind to the W boxes in its promoter and turning on its own expression (Mao et al., 2011). Expression of Arabidopsis WRKY33 is also induced by abiotic stress conditions and by paraquate, which generates ROS in exposed plant cells (Zheng et al., 2006). In plants, autophagy is also induced by a variety of stresses, including nutrient deprivation, drought, salt stress, ROS, and pathogen infection (Liu and Bassham, 2010; Lai et al., 2011b; Zhou et al., 2013). It has also been shown that NADPH oxidase inhibitors block autophagy induction by nutrient starvation and salt stress, indicating that ROS may also function as a signal in induction of autophagy by some environmental stresses (Liu and Bassham, 2010). It is tempting to speculate that multiple stress-initiated pathways may converge to the activation and induction of WRKY33 for induction of some autophagy- and other stress-related genes. Although autophagy is highly conserved in eukaryotic organisms, WRKY transcription factors are mostly plant-specific (Zhang and Wang, 2005). A critical role of WRKY33 in the regulation of plant autophagy genes would strongly indicate that the regulatory mechanisms of autophagy in plants have diverged from those in other eukaryotic organisms.

### Conflict of interest statement

The authors declare that the research was conducted in the absence of any commercial or financial relationships that could be construed as a potential conflict of interest.

## Supplementary material

Supplemental Figure 1 **Gene structures and coding sequences of tomato *ATG5a* and *ATG5b*. (A)** Gene structures of tomato *ATG5a* and *ATG5b*. **(B)** Comparison of tomato *ATG5a* and *ATG5b* nucleotide sequences. Identical nucleotides are in red.

Supplemental Figure 2 **Protein sequence comparison of tomato ATG5a and ATG5b**. Identical amino acid residues between tomato ATG5a and ATG5b are in red.

Supplemental Figure 3 **Protein sequence comparison of tomato NBR1a and NBR1b**. Identical amino acid residues between tomato BNR1a and NBR1b are in red.

Supplemental Figure 4 **Protein sequence comparison of Arabidopsis WRKY33, tomato WRKY33a (Sl06g066370), and WRKY33b (Sl09g014990)**. Amino acid residues of tomato WRKY33a or WRKY33b identical to those of Arabidopsis WRKY33 are in red. The highly conserved WRKYGQK sequences and the residues forming the C_2_H_2_ zinc fingers are in blue.

## References

[B1] Bassham D. C. Laporte M. Marty F. Moriyasu Y. Ohsumi Y. Olsen L. J. (2006). Autophagy in development and stress responses of plants. Autophagy 2, 2–11 16874030 10.4161/auto.2092

[B2] Biederbick A. Kern H. F. Elsasser H. P. (1995). Monodansylcadaverine (MDC) is a specific *in vivo* marker for autophagic vacuoles. Eur. J. Cell Biol. 66, 3–14 7750517

[B3] Chikte S. Panchal N. Warnes G. (2014). Use of LysoTracker dyes: a flow cytometric study of autophagy. Cytometry A 85, 169–178 10.1002/cyto.a.22312 23847175

[B4] Consortium T. T. G. (2012). The tomato genome sequence provides insights into fleshy fruit evolution. Nature 485, 635–641 10.1038/nature11119 22660326 PMC3378239

[B5] Contento A. L. Xiong Y. Bassham D. C. (2005). Visualization of autophagy in Arabidopsis using the fluorescent dye monodansylcadaverine and a GFP-AtATG8e fusion protein. Plant J. 42, 598–608 10.1111/j.1365-313X.2005.02396.x 15860017

[B6] Ekengren S. K. Liu Y. L. Schiff M. Dinesh-Kumar S. P. Martin G. B. (2003). Two MAPK cascades, NPR1, and TGA transcription factors play a role in Pto-mediated disease resistance in tomato. Plant J. 36, 905–917 10.1046/j.1365-313X.2003.01944.x 14675454

[B7] Floyd B. E. Morriss S. C. Macintosh G. C. Bassham D. C. (2012). What to eat: evidence for selective autophagy in plants. J. Integr. Plant Biol. 54, 907–920 10.1111/j.1744-7909.2012.01178.x 23046163

[B8] He C. Klionsky D. J. (2009). Regulation mechanisms and signaling pathways of autophagy. Annu. Rev. Genet. 43, 67–93 10.1146/annurev-genet-102808-114910 19653858 PMC2831538

[B9] Hightower L. E. (1991). Heat shock, stress proteins, chaperones, and proteotoxicity. Cell 66, 191–197 10.1016/0092-8674(91)90611-2 1855252

[B10] Huang J. L. Gu M. Lai Z. B. Fan B. F. Shi K. Zhou Y. H. (2010). Functional analysis of the *Arabidopsis PAL* gene family in plant growth, development, and response to environmental stress. Plant Physiol. 153, 1526–1538 10.1104/pp.110.157370 20566705 PMC2923909

[B11] Johansen T. Lamark T. (2011). Selective autophagy mediated by autophagic adapter proteins. Autophagy 7, 279–296 10.4161/auto.7.3.14487 21189453 PMC3060413

[B12] Kandoth P. K. Ranf S. Pancholi S. S. Jayanty S. Walla M. D. Miller W. (2007). Tomato MAPKs LeMPK1, LeMPK2, and LeMPK3 function in the systemin-mediated defense response against herbivorous insects. Proc. Natl. Acad. Sci. U.S.A. 104, 12205–12210 10.1073/pnas.0700344104 17623784 PMC1924534

[B13] Klionsky D. J. (2005). Autophagy. Curr. Biol. 15, R282–R283 10.1016/j.cub.2005.04.013 15854889

[B14] Kraft C. Peter M. Hofmann K. (2010). Selective autophagy: ubiquitin-mediated recognition and beyond. Nat. Cell Biol. 12, 836–841 10.1038/ncb0910-836 20811356

[B15] Lai Z. Li Y. Wang F. Cheng Y. Fan B. Yu J. Q. (2011a). Arabidopsis sigma factor binding proteins are activators of the WRKY33 transcription factor in plant defense. Plant Cell 23, 3824–3841 10.1105/tpc.111.090571 21990940 PMC3229152

[B16] Lai Z. Wang F. Zheng Z. Fan B. Chen Z. (2011b). A critical role of autophagy in plant resistance to necrotrophic fungal pathogens. Plant J. 66, 953–968 10.1111/j.1365-313X.2011.04553.x 21395886

[B17] Lee S. Lee D. W. Lee Y. Mayer U. Stierhof Y. D. Jurgens G. (2009). Heat shock protein cognate 70-4 and an E3 ubiquitin ligase, CHIP, mediate plastid-destined precursor degradation through the ubiquitin-26S proteasome system in Arabidopsis. Plant Cell 21, 3984–4001 10.1105/tpc.109.071548 20028838 PMC2814507

[B18] Li S. Fu Q. Chen L. Huang W. Yu D. (2011). *Arabidopsis thaliana* WRKY25, WRKY26, and WRKY33 coordinate induction of plant thermotolerance. Planta 233, 1237–1252 10.1007/s00425-011-1375-2 21336597

[B19] Liu Y. Bassham D. C. (2010). TOR is a negative regulator of autophagy in *Arabidopsis thaliana*. PLoS ONE 5:e11883 10.1371/journal.pone.0011883 20686696 PMC2912371

[B20] Liu Y. Bassham D. C. (2012). Autophagy: pathways for self-eating in plant cells. Annu. Rev. Plant Biol. 63, 215–237 10.1146/annurev-arplant-042811-105441 22242963

[B21] Liu Y. Burgos J. S. Deng Y. Srivastava R. Howell S. H. Bassham D. C. (2012). Degradation of the endoplasmic reticulum by autophagy during endoplasmic reticulum stress in Arabidopsis. Plant Cell 24, 4635–4651 10.1105/tpc.112.101535 23175745 PMC3531857

[B22] Liu Y. Schiff M. Dinesh-Kumar S. P. (2002). Virus-induced gene silencing in tomato. Plant J. 31, 777–786 10.1046/j.1365-313X.2002.01394.x 12220268

[B23] Liu Y. Xiong Y. Bassham D. C. (2009). Autophagy is required for tolerance of drought and salt stress in plants. Autophagy 5, 954–963 10.4161/auto.5.7.9290 19587533

[B24] Mao G. Meng X. Liu Y. Zheng Z. Chen Z. Zhang S. (2011). Phosphorylation of a WRKY transcription factor by two pathogen-responsive MAPKs drives phytoalexin biosynthesis in Arabidopsis. Plant Cell 23, 1639–1653 10.1105/tpc.111.084996 21498677 PMC3101563

[B25] Munafo D. B. Colombo M. I. (2001). A novel assay to study autophagy: regulation of autophagosome vacuole size by amino acid deprivation. J. Cell Sci. 114, 3619–3629 11707514 10.1242/jcs.114.20.3619

[B26] Otegui M. S. Noh Y. S. Martinez D. E. Vila Petroff M. G. Staehelin L. A. Amasino R. M. (2005). Senescence-associated vacuoles with intense proteolytic activity develop in leaves of Arabidopsis and soybean. Plant J. 41, 831–844 10.1111/j.1365-313X.2005.02346.x 15743448

[B27] Phadwal K. Alegre-Abarrategui J. Watson A. S. Pike L. Anbalagan S. Hammond E. M. (2012). A novel method for autophagy detection in primary cells: impaired levels of macroautophagy in immunosenescent T cells. Autophagy 8, 677–689 10.4161/auto.18935 22302009 PMC3405842

[B28] Shaid S. Brandts C. H. Serve H. Dikic I. (2013). Ubiquitination and selective autophagy. Cell Death Differ. 20, 21–30 10.1038/cdd.2012.72 22722335 PMC3524631

[B29] Slavikova S. Ufaz S. Avin-Wittenberg T. Levanony H. Galili G. (2008). An autophagy-associated Atg8 protein is involved in the responses of Arabidopsis seedlings to hormonal controls and abiotic stresses. J. Exp. Bot. 59, 4029–4043 10.1093/jxb/ern244 18836138 PMC2576633

[B30] Svenning S. Lamark T. Krause K. Johansen T. (2011). Plant NBR1 is a selective autophagy substrate and a functional hybrid of the mammalian autophagic adapters NBR1 and p62/SQSTM1. Autophagy 7, 993–1010 10.4161/auto.7.9.16389 21606687 PMC3210314

[B31] Xiong Y. Contento A. L. Bassham D. C. (2007a). Disruption of autophagy results in constitutive oxidative stress in Arabidopsis. Autophagy 3, 257–258 17312382 10.4161/auto.3847

[B32] Xiong Y. Contento A. L. Nguyen P. Q. Bassham D. C. (2007b). Degradation of oxidized proteins by autophagy during oxidative stress in Arabidopsis. Plant Physiol. 143, 291–299 10.1104/pp.106.092106 17098847 PMC1761971

[B33] Yoshimoto K. (2010). Plant autophagy puts the brakes on cell death by controlling salicylic acid signaling. Autophagy 6, 192–193 10.4161/auto.6.1.10843 20023431

[B34] Zhang Y. Wang L. (2005). The WRKY transcription factor superfamily: its origin in eukaryotes and expansion in plants. BMC Evol. Biol. 5:1 10.1186/1471-2148-5-1 15629062 PMC544883

[B35] Zheng Z. Qamar S. A. Chen Z. Mengiste T. (2006). Arabidopsis WRKY33 transcription factor is required for resistance to necrotrophic fungal pathogens. Plant J. 48, 592–605 10.1111/j.1365-313X.2006.02901.x 17059405

[B36] Zhou J. Wang J. Cheng Y. Chi Y. J. Fan B. Yu J. Q. Chen Z. (2013). NBR1-Mediated selective autophagy targets insoluble ubiquitinated protein aggregates in plant stress responses. PLoS Genet. 9:e1003196 10.1371/journal.pgen.1003196 23341779 PMC3547818

[B37] Zhou J. Xia X. J. Zhou Y. H. Shi K. Chen Z. Yu J. Q. (2014a). *RBOH1*-dependent H_2_O_2_ production and subsequent activation of MPK1/2 play an important role in acclimation-induced cross-tolerance in tomato. J. Exp. Bot. 65, 595–607 10.1093/jxb/ert404 24323505 PMC3904713

[B38] Zhou J. Yu J. Q. Chen Z. (2014b). The perplexing role of autophagy in plant innate immune responses. Mol. Plant Pathol. [Epub ahead of print]. 10.1111/mpp.12118 24405524 PMC6638830

[B39] Zhou J. Zhang Y. Qi J. Chi Y. Fan B. Yu J. Q. (2014c). E3 ubiquitin ligase CHIP and NBR1-mediated selective autophagy protect additively against proteotoxicity in plant stress responses. PLoS Genet. 10:e1004116 10.1371/journal.pgen.1004116 24497840 PMC3907298

