# Supplementary material for: Role and regulation of autophagy in heat stress responses of tomato plants
Source: Front Plant Sci. 2014 Apr 30;5:174. doi: 10.3389/fpls.2014.00174 (PMC4012191; doi:10.3389/fpls.2014.00174)
Supplement: Supplemental Figure 1 — Gene structures and coding sequences of tomato ATG5a and ATG5b. (A) Gene structures of tomato ATG5a and ATG5b. (B) Comparison of tomato ATG5a and ATG5b nucleotide sequences. Identical nucleotides are in red. [file DataSheet1.PDF]

A

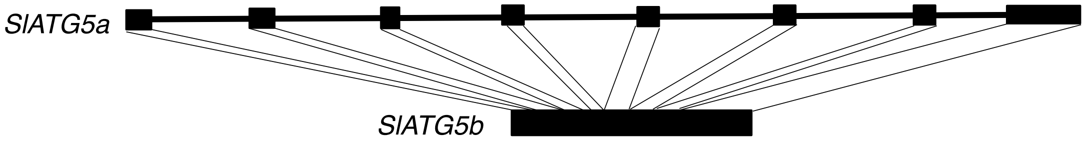

B

SlATG5a ATGGAAAGTAAAGGAGGAGGAACTGAAGCACAGAAGTATATATGGGAAGGAGCAATCCCT 60
SlATG5b ATGGAAAGTAAAGGAGGAGGAACTGAAGCACAGAAGTATGTATGGGAAGGAGCAATCCCT 60

SlATG5a CTGCAGATTCATCTTCATGAATCCGAAATCACTACTCTCCCTACTCCTCCCCCTGCTATG 120
SlATG5b CTGCAGATTCATCTTCATGAATCCAAAATGACTACTCTCCCTACTCCTCCCCCTGCTATG 120

SlATG5a ATTTTAGCTCCTCGAATTGGGTACCTGCCTCTTTTAGCACAAAAAGTAAAGCCTTTCTTC 180
SlATG5b ATTTTAGCTCCTCGAATTGGGTACCTGCCTCTTTTAGCACAAAAAGTAAAGCCTTTCTTC 180

SlATG5a AGTAATTCACTTCCTCCAGGTGTAGATACCATATGGTTTGAGTACAATGGCTTGCCTCTC 240
SlATG5b AGTAATTCACTTCCTCCAGGTGTAGATACGATATGGTTTGAGTACAATGGCTTGCCTCTC 240

SlATG5a AAATGGTATATACCTACTGGGGTACTCTTTGATCTTCTTTGTGCAGAACCTGAACGGCCT 300
SlATG5b AAATGATATATACCTACTGGGGTAATCTTTGATCTTCTTTGTGCAGAACCTGAACGGCCT 300

SlATG5a TGGAATCTGACGGTACATTTTAGAGGATATCCTGGAAATATTTTAACACCCTGTGACAGT 360
SlATG5b TGGAATCTGACAGTACATTTTAGAGGATATCCTGGAAATATTTTAACACCCTGTGACAGT 360

SlATG5a GAAGATAGTGTAAAATGGAGCTTTATCAATTCATTTAAAGAGGCAGCATATATAATCAAT 420
SlATG5b GAAGATAGTGTAAAATGGAGCTTTATTAATTCATTTAAAGAGGCAGCATATATAATCAAT 420

SlATG5a GGGAACTGCAAAAATGTAATGAATATGTCCCAATCTGACCAATTAGAACTCTGGCGCTCC 480
SlATG5b GGGAACTGCAAAAATGTAATGAATATGTCCCAATCTGACCAATTAGAACTCTGGCGCTCC 480

SlATG5a ATTATGGATGGTGATTTGGACTCTTATCTTCGAATCTCATCTAAGCTAAAATTCGGCATA 540
SlATG5b ATTATGGATGGTGATTTGGACTCTTATCTTCGAATCTCATCTAAGCTAAAATTCAGCATT 540

SlATG5a CTTGTGGATGATTTTTCGATACAATTGAACATTTCCTCTCCTAAATCACCGGAAAGCACT 600
SlATG5b CTTGTGGATGATTTTTTGATAAAATTGAACATTTCCTCTCCTAAATCACCTGAAAGCACT 600

SlATG5a CAAAATGCAGATGGCACAGCACCAGCTAAAACTGGTAGAATACCGGTTCGACTGTATGTT 660
SlATG5b CAAAATGCAGATGGCACAGCACCAGCTAAAACTGGTAGAATACCGGTTCGACTGTATGTT 660

SlATG5a AGAACTATCAATGAGGATTTTGACGAGTTAGAAGATGCACCTGTTGTTGAAAGTTGGGAC 720
SlATG5b AGAACTATCAATGAGGATTTTGACGAGTTAGAAGATGCACCTGTTGTTGAAAGTTGGGAC 720

SlATG5a AGAATCTCTTACATAAACAGACCTGTTCAGATCCATGGAGATGGTAAATGCTTCACCTTG 780
SlATG5b AAAATCTCTTACATAAACAGACCTGTTCAGATCCATGGAGATGGTAAATGCTTCACCTTG 780

SlATG5a TATGATGCAGTAACAAAACTTCTGCCCGAGTTTTTTGGGGAAAAACTTCCGCCAACAGAT 840
SlATG5b TCTGATGCAGTAACAAAACTTCTGCCAGAGTTTTTTGGGGAAAAACTTCCGCCAACAGAT 840

SlATG5a GATGTATCTAAAGAAGAAGTTGAGGTTGAACAGAGATCATCTCCAGAAGAAACAAACAAG 900
SlATG5b GATGTATCTAAAGAAGAAGTTGAGATTGAACAGAGATCATCTCTAGAAGAAACAAACAAG 900

SlATG5a AGCAACACGGAACGAAGTGGGGAGATGTTGAATGAGGGCATCGTCTCTTGCTCTATTTCA 960
SlATG5b AGCAACACGGAACGAAGTGGGGAGATGTTGAATGAGGGCATCGTCGCTTGCTCTATTTCA 960

SlATG5a GATGGTGCTGAGATCAAGCTCCTGCGCATTCAGGGAATTGAACCAAAGATGGAGATTCCT 1020
SlATG5b GATGGTGCTGAGATCAAGCTCCTGCGCATTCAGGGAATTGAACCAAAGATGGAGATTCCT 1020

SlATG5a TTTGCATGGGTGGTAAACAATTTGATGAACCCTGAGTACTTTCTTCATATTTGTGTGTAT 1080
SlATG5b TTTGCATGGGTGGTAAACAATTTGATGAACCCTGAGTACTTTCTTCATATTTGTGTGTGA 1080

Supplemental Figure 2

```
SlATG5a  1    MESKGGGTEAQKYIWEGAIPLQIHLHESEITTLPTPPPAMILAPRIGYLPLLAQKVKPFF  60
SlATG5b  1    MESKGGGTEAQKYVWEGAIPLQIHLHESKMTTLPTPPPAMILAPRIGYLPLLAQKVKPFF  60

SlATG5a  61   SNSLPPGVDTIWFEYNGLPLKWYIPTGVLFDLLCAEPERPWNLTVHFRGYPGNILTPCDS  120
SlATG5b  61   SNSLPPGVDTIWFEYNGLPLK*YIPTGVIFDLLCAEPERPWNLTVHFRGYPGNILTPCDS  120

SlATG5a  121  EDSVKWSFINSFKEAAYIINGNCKNVMNMSQSDQLELWRSIMDGDLDSYLRISSKLKFGI  180
SlATG5b  121  EDSVKWSFINSFKEAAYIINGNCKNVMNMSQSDQLELWRSIMDGDLDSYLRISSKLKFSI  180

SlATG5a  181  LVDDFSIQLNISSPKSPESTQNADGTAPAKTGRIPVRLYVRTINEDFDELEDAPVVESWD  240
SlATG5b  181  LVDDFLIKLNISSPKSPESTQNADGTAPAKTGRIPVRLYVRTINEDFDELEDAPVVESWD  240

SlATG5a  241  RISYINRPVQIHGDGKCFTLYDAVTKLLPEFFGEKLPPTDDVSKEEVEVEQRSSPEETNK  300
SlATG5b  241  KISYINRPVQIHGDGKCFTLSDAVTKLLPEFFGEKLPPTDDVSKEEVEIEQRSSLEETNK  300

SlATG5a  301  SNTERSGEMLNEGIVSCSISDGAEIKLLRIQGIEPKMEIPFAWVVNNLMNPEYFLHICVY  359
SlATG5b  301  SNTERSGEMLNEGIVACSISDGAEIKLLRIQGIEPKMEIPFAWVVNNLMNPEYFLHICV*  359

SlATG5a  360  YVKIQEPITI
SlATG5b  360
```

Supplemental Figure 3

```
SlNBR1a   1   MAMESSIVIKVKYEETLRRFNACVINEKLDLDIGGLRDKIIRLFNFAHDAEITLTYIDEDGD   62
SlNBR1b   1     MESSIVIKVKYGETLRRFNARVADDKLGLNIDGLKDKIFKLFNFPPDSELTLTYIDEDGD   60

SlNBR1a   123   SRVSDVLKYIPEPLRESVMKVCSDVTASASSSAPILAELVDAMCELGLSHYQNQVSGPQP   182
SlNBR1b   117   SSVSDLLKSLPKSKSKKILKHSADMASKASSAAREIAELSKALSVTSLSY----------   166

SlNBR1a   183   VKEAGSCSGISKGNAMSADGVMPNVKIGESSAKKNGPLTAVHGEEPTLKTTEPKPNASNA   242
SlNBR1b   167   LKQACPVSGVPMGS----------VKSGE-------PSQAANPEELTVKTAG-RPKSHTV   208

SlNBR1a   243   AVDASVKLVSKSETLEG------DRTKAQSSSFEASKAQKDKKFDVRSLDGRTIGYGYAR   296
SlNBR1b   209   SINAS-ELKSSQPDQNGIQCEPLSKSPKRNSSLVDGKKEEGNKFGDSHLVGKALGNSDPS   267

SlNBR1a   297   NSPIPPEKTSDEQPSKGHPVAKPVDLGGSASSSNVKQCNWDSLNADSSGSLINIPYDGFT   356
SlNBR1b   268   ASTTGPKKTADKQQTENHP--------------------------------------GAE   289

SlNBR1a   357   PSHVVHLNTVNVNDSHNAGSSGSSMKMPYDGYIPAVRHLGPLIPVNACPFSGMPTENNPI   416
SlNBR1b   290   PVGVVGL----------SGKLSGGFRSPISYWMPMV-----------------PVSNDTI   322

SlNBR1a   417   PPQNFSFEVPLKRSHNHSDGTGTIFHKGVRCDGCGVHPITGPRFISKVKENYDLCSICFA   476
SlNBR1b   323   QPQYSTFRIPVKRSHNHSDGTGSIFHRGVRCDGCGVHPITGPRFKSKVKEDYDLCSICFA   382

SlNBR1a   477   EMGNDADYFRMDRPLTYPHPWSFKGLHDLHGRLRPRPPTVPQVIRGFGLKAGRPKLDSRF   536
SlNBR1b   383   QMGIDADYVRMDRPVSYHHPIAFKALHEPH-----------DIFRGCGVKS--PKLDSRF   429

SlNBR1a   537   IQDVNVLDGTIMAPLTQFTKIWRMKNNGNLVWPQGTQLVWIGGDKLSDRFSVELEMTTAG   596
SlNBR1b   430   KHDVNVLDGTMMAPSTPFTKVWRMRNNGNIFWPQGTQLVWIGGDRLGDAVSVELQIPSFG   489

SlNBR1a   597   LAVDQELDVAVDFAAPEHPGRYISYWRLASPSGQKFGQRVWVLIQVDALLSLPKRGLVHE   656
SlNBR1b   490   LAVDHEFDVAVDFRAPKLPGRYISFWRMALPSGEKFGQRVWVLIQVDFSM-IPKKEFSYE   548

SlNBR1a   657   AFQGLNLNLPPASSGVSGADIINVNSEP--HNVVPEPKSSN-TMELVDSVAEVNQNMEQE   713
SlNBR1b   549   ASQVLDLNLPPAGYDIAGSEYINVNADMTIEDIIADPKISNPATGSVEPVVDGNRNNE-E   607

SlNBR1a   714   VKFPINDSLLVGFGDKSSSPSASGSTISYPIIDLTKEPPSEDSSMQPSAVVAMQAPPLQD   773
SlNBR1b   608   FKSCI-------------SPSAAGSSISYP-IDLSEAAP-EVTSVAPPSVVEVQASPQED   652

SlNBR1a   774   ARGNFEVETSLLQELEEMGFKQVDLNKEILRKNEYDLEQSVDDLCGVAEWDPILEELKDM   833
SlNBR1b   653   ------VEMSLLKELDDMGFTQVNLNKEVLRMNEYNLEQSVADLCGVSEWDPILEELEEM   706

SlNBR1a   834   GFCNKEMNKKLLKKNNGSIKRVVMDLIAGEQ   864
SlNBR1b   707   GFHNKEINKTLLKKNNGSIKRVVMDLIAGEN   737
```

Supplemental Figure 4

```
AtWRKY33    MAAS------FLTMDNS-RTRQNMNGSANWSQQSGRTSTSSLED-------LEIPKFRSF
SlWRKY33a   MASSGGNMNTFMNSFNSFSSSQFMTSSFSDLLSDNNNNNNSNND----NKNWGFSEDRSK
SlWRKY33b   MAASS-----FSFPTSSSS---FMTTSFTDLLASDDYPTKGLADRIAERTGSGVPKFKSL

AtWRKY33    APSSISISPSLVSPS------TCFSPSLFLDSPAFVSSSANVLASPTTGALITNVTNQKG
SlWRKY33a   SFPMMNSSTSPASPSSYLAFPHSLSPSMLLDSPVLFNNS-NTLSSPTTGSFGN--LNSK-
SlWRKY33b   PPPSLPLSPPPPFSPSSYFAIPPGLSPTELLDSPVLLSSS-NLLPSPTTGSFPSRAFNWKS

AtWRKY33    INEGD----KSNNNNNFNLFDFSFHTQSSGVSAPTTTT----TTTTTTTTTNSSIFQSQEQ
SlWRKY33a   ----------EGNSEFSFQSRP-ATSSSIFQSSAPRNSLEDLMTRQQQTTEFSTAKTGVK
SlWRKY33b   SSHQDVKQEDKNYSDFSFQPQVGTAASSISQSQTNHVPLGQQAWNCQEPTKQNDQNANGR

AtWRKY33    QKKNQSEQWSQTETRPNNQAVSYNG-----REQRKGEDGYNWRKYGQKQVKGSENPRSYY
SlWRKY33a   SEVAPIQSFSQENMSNNPAPVHYCQPSQYVREQK-AEDGYNWRKYGQKQVKGSENPRSYY
SlWRKY33b   SEFNTVQNFMQNNNDQNNSGNQYNQS---IREQKRSDDGYNWRKYGQKQVKGSENPRSYY

AtWRKY33    KCTFPNCPTKKKVERSLEGQITEIVYKGSHNHPKPQSTRRSSSSSTFHSAVYNASLDHN
SlWRKY33a   KCTFPNCPTKKKVERNLDGHITEIVYKGSHNHPKPQSTRRSSSQS---IQNLAYSNLDVT
SlWRKY33b   KCTYPNCPTKKKVERSLDGQITEIVYKGNHNHPKPQSTRRSSSST---ASSAFQSYNTQT

AtWRKY33    RQASSDQPNSNNSFHQSDSFGMQQEDNTTSDSVGDDE---FEQGSSIVSRDEEDCGSEPE
SlWRKY33a   NQP---NAFLEN--GQRDSFAVTD---NSSASFGDDD---VDQGSPISKSGENDE-NEPE
SlWRKY33b   NEIPDHQSYGSN--GQMDSVATPE---NSSISFGDDDHEHTSQKSSRSRGDDLDE-EEPD

AtWRKY33    AKRWKGDNETNGGNG-GGSKTVREPRIVVQTTSDIDILDDGYRWRKYGQKVVKGNPNPRS
SlWRKY33a   AKRWKGDNENEVISS--ASRTVREPRIVVQTTSDIDILDDGYRWRKYGQKVVKGNPNPRS
SlWRKY33b   SKRWKRENESEGVSALGGSRTVREPRVVVQTTSDIDILDDGYRWRKYGQKVVKGNPNPRS

AtWRKY33    YYKCTTIGCPVRKHVERASHDMRAVITTYEGKHNHDVPAARGSG-YATNRAP-----QDS
SlWRKY33a   YYKCTFTGCPVRKHVERASHDLRAVITTYEGKHNHDVPAARGSGSYAMNKPPSGSNNNNS
SlWRKY33b   YYKCTSTGCPVRKHVERASQDIRSVITTYEGKHNHDVPAARGSGNHSINRP---------

AtWRKY33    SSVPIRPAAIAGHSNY----------TTSSQAPYTLQMLHNNNTNTGPFGYAMNNNNNNS
SlWRKY33a   MPVVPRPTVLANHSNQGMNFNDTFFNTTQIQPPITLQMLQSSGT-SSYSGFG-NSSGSYM
SlWRKY33b   MAPTIRPTVTSHQSNYQVPLQSIRPQQSEMGAPFTLEMLQKPNN-YGFSGYA-NSGDSYE

AtWRKY33    NL-QTQQNFVGGGFSRAKEEPNEETSFFDSFMP
SlWRKY33a   NQMQHTNNSK----PISKEEPKDD-LFFSSFLN
SlWRKY33b   NQVQD-NNVF----SRTKDEPRDD-LFMESLLC
```
