# Supplementary material for: Role and regulation of autophagy in heat stress responses of tomato plants
Source: Front Plant Sci. 2014 Apr 30;5:174. doi: 10.3389/fpls.2014.00174 (PMC4012191; doi:10.3389/fpls.2014.00174)
Supplement: Supplementary file 2 [file DataSheet2.PDF]

Supplemental Table 1: Primers for qRT-PCR

Name	Gene identifier	Primer
ATG5	Sl02g036380	F: 5′ TCAGATGGTGCTGAGATCAAG 3′<br>R: 5′ ATTGTTTACCACCCATGCAA 3′
ATG7	Sl11g068930	F: 5′ ATTCAACGGCTAACCGTACC 3′<br>R: 5′ CAAACTCAGCTTTGGCACAT 3′
NBR1a	Sl03g112230	F: 5′ CTCCATCTGCTTCTGGTTCA 3′<br>F: 5′ TGATGGTTGCATGGAAGAGT 3′
NBR1b	Sl06g071770	F: 5′ CAAGGAACTGGATGACATGG 3′<br>F: 5′ GCCATTGTTCTTCTTGAGCA 3′
WRKY33a	Sl09g014990	F: 5′ GCATTACTGTCAACCATCGC 3′<br>R: 5′ AACTTCGCGGATTCTCACTT 3′
WRKY33b	Sl06g066370	F: 5′ CCACAACAGTCTGAAATGGG 3′<br>R: 5′ CAGCAAAGCAATGACTCCAT 3′
HSP17.6	Sl08g062340	F: 5′ GAGAAGGGTTGGGAAATTCA 3′<br>R: 5′ ACTTCTTTGGCTCAGGAGGA 3′
HSP20	Sl01g102960	F: 5′ GTTCCCACAAGAAGACCCAT 3′<br>R: 5′ TCCCAGGATGTCAAGTGAA 3′
HSP40	Sl01g057650	F: 5′ TTGACAAGGACAAGGACGAG 3′<br>R: 5′ CATTTAGTGGCTCTGGCTCA 3′
HSP100	Sl02g088610	F: 5′ GTTAGGCCGAGAGTTGGAAG 3′<br>R: 5′ TCTTGAATGAAGCCAAGCAC 3′
Actin	Sl03g078400	F: 5′ TGTCCCTATTTACGAGGGTTATGC 3′<br>R: 5′ CAGTTAAATCACGACCAGCAAGAT 3′

Supplemental Table 2. Primers for generating VIGS constructs.

Name	Gene identifier	Primer
ATG5	Sl02g036380	5′CGACGACAAGACCCTAACGGCCTTGGAATCTGAC3′<br>5′GAGGAGAAGAGCCCTTCTCCATGGATCTGAACAGG3′
ATG7	Sl11g068930	5′CGACGACAAGACCCTCAAGGAGGGAGGAATCATCA3′<br>5′GAGGAGAAGAGCCCTATGTCCAGGCATCGGAATAG3′
NBR1a	Sl03g112230	5′CGACGACAAGACCCTCCAAAACCTAATGCCTCCAA3′<br>5′GAGGAGAAGAGCCCTAGCCATCATACGGCATCTTC3′
NBR1b	Sl06g071770	5′CGACGACAAGACCCTGCGTCTAAAGCCTCATCAGC3′<br>5′GAGGAGAAGAGCCCTGCACCAGGGTGATTCTCAGT3′
WRKY33a	Sl09g014990	5′ATCGAATTCCCATTGCAGTCTTGTATCT3′<br>5′ATCCTCGAGTGTTTTGTGGGCTCTTGACA3′
WRKY33a	Sl09g014990	5′ATCGAATTCCATCTGGAAGCAACAACA3′<br>5′ATCCTCGAGCATGAAAACTCAGTTCCACCT3′
